# Regulation of PTEN/Akt Pathway Enhances Cardiomyogenesis and Attenuates Adverse Left Ventricular Remodeling following Thymosin β4 Overexpressing Embryonic Stem Cell Transplantation in the Infarcted Heart

**DOI:** 10.1371/journal.pone.0075580

**Published:** 2013-09-24

**Authors:** Binbin Yan, Reetu D. Singla, Latifa S. Abdelli, Pawan K. Singal, Dinender K. Singla

**Affiliations:** 1 Burnett School of Biomedical Sciences, College of Medicine, University of Central Florida, Orlando, Florida, United States of America; 2 Institute of Cardiovascular Sciences, St. Boniface General Hospital Research Centre, University of Manitoba, Winnipeg, Manitoba, Canada; University of Texas Medical Branch, United States of America

## Abstract

Thymosin β4 (Tβ4), a small G-actin sequestering peptide, mediates cell proliferation, migration, and angiogenesis. Whether embryonic stem (ES) cells, overexpressing Tβ4, readily differentiate into cardiac myocytes *in vitro* and *in vivo* and enhance cardioprotection following transplantation post myocardial infarction (MI) remains unknown. Accordingly, we established stable mouse ES cell lines, RFP-ESCs and Tβ4-ESCs, expressing RFP and an RFP-Tβ4 fusion protein, respectively. *In vitro*, the number of spontaneously beating embryoid bodies (EBs) was significantly increased in Tβ4-ESCs at day 9, 12 and 15, compared with RFP-ESCs. Enhanced expression of cardiac transcriptional factors GATA-4, Mef2c and Txb6 in Tβ4-EBs, as confirmed with real time-PCR analysis, was accompanied by the increased number of EB areas stained positive for sarcomeric α-actin in Tβ4-EBs, compared with the RFP control, suggesting a significant increase in functional cardiac myocytes. Furthermore, we transplanted Tβ4-ESCs into the infarcted mouse heart and performed morphological and functional analysis 2 weeks after MI. There was a significant increase in newly formed cardiac myocytes associated with the Notch pathway, a decrease in apoptotic nuclei mediated by an increase in Akt and a decrease in levels of PTEN. Cardiac fibrosis was significantly reduced, and left ventricular function was significantly augmented in the Tβ4-ESC transplanted group, compared with controls. It is concluded that genetically modified Tβ4-ESCs, potentiates their ability to turn into cardiac myocytes *in vitro* as well as *in vivo*. Moreover, we also demonstrate that there was a significant decrease in both cardiac apoptosis and fibrosis, thus improving cardiac function in the infarcted heart.

## Introduction

Embryonic stem (ES) cells, derived from early embryos, have the potential to self-renew and differentiate into any cell type of all three primary germ layers: ectoderm, endoderm, and mesoderm [1,2]. Previous studies have demonstrated that ES cells can differentiate into a variety of specialized cell types including cardiac myocytes both *in vitro* and *in vivo* [3-5]. However, the number of ES cell derived cardiac myocytes is very limited in the absence of cell culture or genetic modifications. Discovery of human ES cells and their potential to differentiate into cardiac myocytes has provided a foundation for future explorations to identify various growth factors and signaling molecules that enhance cardiac myocyte differentiation. Recent stem cell therapy studies suggest that ES cells may provide a new approach to repair injured myocardium. Indeed, transplanted ES cells in the infarcted mouse and rat hearts can differentiate into cardiac myocytes, contributing to improved cardiac function [6-8]. However, data obtained from these studies raise major concerns regarding the limited capacity of cardiac myocyte differentiation following ES cell transplantation. In this regard, identification of appropriate factors, which will enhance cardiac myocyte differentiation from ES cells, has gained recent significant attention. In fact, various factors such as TGFβ2 [9], BMP4 [10], vitamin C [11], etc. have been reported to promote cardiac myocyte differentiation from ES cells. Nevertheless, there has been increased interest in search of novel cardiomyogenic factors.

Thymosin β4 (Tβ4), a highly conserved, 43-amino acid peptide, is the most abundant member of the β–thymosin family in a majority of mammalian tissues [12-14]. Tβ4 has been shown to play an important role in the regulation of cell proliferation, migration, and angiogenesis [15-17]. Recently, Tβ4 has been found to protect against myocardial injury via inhibiting apoptosis and reducing infarct size with improved myocardial function [15,16,18,19]. Moreover, knockdown of Tβ4 by shRNA deprives embryonic endothelial progenitor cells of their capacity to preserve myocardial function in the ischemic heart [20]. In contrast, Tβ4 overexpression in endothelial cells inhibits apoptosis after hypoxia-reoxygenation and also decreases the adhesion of inflammatory cells to an endothelial cell layer *in vitro* [20]. Furthermore, Tβ4 stimulates the migration of hair follicle stem cells, resulting in hair growth [21], and promotes differentiation of epicardial progenitor cells to vascular cells [16]. However, there is no information available regarding the role of Tβ4 in cardiac myocyte differentiation from ES cells. In the present study, we examined the effects of Tβ4 on cardiac myocyte differentiation using a genetically modified ES cell line (Tβ4-ESCs) and further investigated whether or not Tβ4-ESCs transplanted in the mouse heart produce cardioprotective effects following myocardial infarction.

## Materials and Methods

### Cell Culture

Undifferentiated mouse ES cells were cultured in the absence of feeder cells on 0.1% gelatin coated plates in growth medium consisting of DMEM supplemented with 0.2 mM L-glutamine, 0.1 mM β-mercaptoethanol, 0.1 mM nonessential amino acids, 10% fetal bovine serum (FBS), leukemia inhibitory factor (LIF, 2000U/ml), 50U/ml penicillin, and 50 µg/ml streptomycin, as we reported previously [9].

### Construction of Expression Vectors

The mammalian expression vector (pTurboFP635-C) encoding red fluorescent protein (RFP) was purchased from Evrogen (Moscow, Russia). Mouse Tβ4 gene was generated by PCR using high fidelity DNA polymerase and the primers containing Bgl II and BamH I sites (forward: 5’-AGATCTAAAGTCCAAGGCATGCTCC-3’ and reverse: 5’-GGATCCTTACGATTCGCCA GCTTG-3’). The PCR product was cloned to the vector pTurboFP635 along with the antibiotic blasticidin resistance gene (Invitrogen Life Technologies, Carlsbad, CA). Restriction enzyme digestion and DNA sequencing was used to confirm the sequence of the mouse Tβ4 gene.

### Transfection and Generation of Stable Cell Lines

ES cells were cultured in 6-well plates at a density of 20,000 cells per well. Cells were transfected with or without Tβ4 expressing vectors using the lipofectamine 2000 (Invitrogen Life Technologies) according to the manufacturer’s instructions. Stably transfected cells were selected using the antibiotic blasticidin. Cells were selected with 2 µg/ml blasticidin for 3 weeks and were monitored daily by fluorescence microscopy. Transfected cells containing blasticidin resistance gene were unaffected, whereas untransfected cells were killed during drug selection. Surviving transfected cell clones expressing RFP were identified by fluorescent microscopy. Following identification of stably transfected ES cells, Tβ4 expressing ES cells were maintained in a routine cell culture and used for future studies.

### Differentiation of ES Cells into Cardiac Myocytes

ES cells with or without Tβ4 overexpression were used for cardiac myocyte differentiation. Differentiation of ES cells was initiated using the hanging drop method, as described previously [22]. In brief, hanging drops (~500 cells in 30 µl of differentiation medium) were generated to form embryoid bodies (EBs). The differentiation medium consisted of DMEM supplemented with 0.2 mM glutamine, 0.1mM β-mercaptoethanol, 0.1 mM nonessential amino acids and 15% FBS, but without LIF. At day 3 (D3), the EBs were transferred to 100 mm Petri dishes for 2 days. At D5, the EBs were plated on 0.1% gelatinized plates (~30-40 EBs per 100 mm dish, ~2 EBs on a 12-well plates) and observed for beating EBs. The medium was changed every 2 days. The morphological changes and spontaneously beating areas in the differentiating EBs were examined by light and fluorescent microscopy at D9, D12 and D15.

### Western Blot Analysis

EBs were washed with phosphate-buffered saline (PBS) and lysed in RIPA buffer containing 150 mM NaCl, 10 mM Tris-HCl (pH 7.4), 1 mM EDTA, 1% Triton X-100, 1% deoxycholic acid, 1 mM PMSF, and 10 mg/ml each of leupeptin and aprotinin. The whole cell lysates (20-50 µg of protein) from each sample were separated by electrophoresis on a 10% polyacrylamide gel. The size-fractionated proteins were transferred to PVDF membranes and blocked with a buffer containing 5% non-fat milk and 0.1% Tween-20 in PBS for 2 hours. Goat anti-Tβ4 and mouse anti-sarcomeric α-actin antibodies were diluted (1:1,000) in blocking buffer, and membranes were incubated with antibodies at 4°C for 24 hours. After incubation, the membranes were washed 3 times with Tris-Buffered Saline with Tween-20 (TBST) containing 10 mM NaCl, 10 mM Tris-HCl and 0.1% Tween-20, followed by incubation with horseradish peroxidase-conjugated anti-goat and anti-mouse secondary antibodies at room temperature for 1 hour. The membranes were washed with TBST buffer. The immune complexes were developed by enhanced chemiluminescence and visualized after exposure to Kodak x-ray film.

### Real-Time PCR

Total RNA was extracted from ES cells with Tri-reagents (Sigma), and samples were treated with DNase I (Ambion) to prevent DNA contamination. cDNA was synthesized from 1 µg total RNA using Superscript II reverse transcriptase and random primers (Bio-Rad). Real-time PCR was performed using a Bio-Rad I-cycler and master SYBR green I dye mix (Bio-Rad). The following cycling conditions were used: 95°C for 5 min followed by 35 cycles of amplification (95°C denature for 15 seconds and 60°C for combined annealing/extension for 60 seconds). PCR primers were synthesized as follows: GATA-4 forward 5’- GGTTCC CAG GCCTCT TGC AAT GCG G -3’ and reverse 5- AGT GGC ATT GCT GGA GTT ACC GCT G -3, Mef2c forward 5’- AGATAC CCA CAA CAC ACC ACG CGC C and reverse 5’- ATC CTT CAG AGA GTC GCATGC GCT T -3’, Tbx6 forward 5’- AGC CAC CCA ACT ATG CAG CCA ACA C -3 and reverse 5’ - GCATCC CGCTCC CTC TTA CAGTTT C -3’, and β-tubulin forward 5’-CCGGACAGTGTGGCAACCAGATCGG-3’ and reverse 5’-TGGCCAAAAGGACCTGAGC GAACGG -3’. All reactions were performed in duplicate. The level of cardiac gene expression was normalized to the expression level of the housekeeping gene tubulin.

### Immunocytochemistry and Immunohistochemistry

EBs or digested EBs with Cellutron enzyme solution (Cellutron Life Technologies) were washed with PBS and fixed with 4% paraformaldehyde for 15 minutes at room temperature. Non-specific blocking was performed by incubation for 1 hour with blocking buffer containing 2% BSA, 0.1% Triton X100 in PBS. After incubation, three washings were given with PBS. Next, EBs were incubated with primary antibodies such as anti-mouse sarcomeric α-actin (S-Actin, Sigma) and anti-Tβ4 at 4 °C for 24 hours. EBs were washed and then incubated with fluorescein-conjugated anti-mouse secondary antibodies (Vector Labs, CA) for 1 hour at room temperature. EBs were mounted with Vectashield medium containing 4'-6-diamidino-2-phenylindole (DAPI, Vector Labs, Burlingame, CA) to visualize nuclei. Stained cells were analyzed by fluorescence (Olympus) or confocal microscopy. For immunohistochemical staining, heart tissues were fixed in 4% buffered formalin, embedded in paraffin, and cut into 5 µm serial sections. The heart sections were then deparaffinized, rehydrated, and stained with Masson’s trichrome for visualization of interstitial fibrosis. Interstitial fibrotic areas (with collagen stained blue) were measured within the infarct, peri-infarct and non-infarct regions and calculated using ImageJ software (NIH).

### Determination of MMP-9 Activity

MMP-9 activity was examined in control and experimental groups by using a commercially available mouse MMP-9 kit (R&D Systems, Minneapolis, MN). Isolated heart tissue was homogenized and supernatant was used to perform MMP-9 assay as per instructions in the kit. The developed reaction was read at 450 nm using a BioRad plate reader. The examined optical density was normalized to the previously determined protein concentration in the samples and data was plotted as A.U.

### Mouse MI Model and Cell Transplantation

All animals were maintained and used as approved by the University of Central Florida animal review board. MI was performed in C57BL/6 mice (Jackson laboratories) by permanent ligation of left coronary artery as described previously [23-25]. In brief, mice were divided into four experimental groups: Sham, MI, MI + RFP-ES cells and MI + Tβ4 -ES cells. Mice were anesthetized with 2.5% isoflurane, intubated, and ventilated using a rodent MiniVent (Harvard Apparatus). A left thoracotomy was performed and the left anterior descending (LAD) coronary artery was ligated with a 7-0 ligature (CP Medical). Following ligation, 2 intramyocardial injections into the peri-infarct region were given containing total 5x10^4^ cells in 20 µl media. Sham mice received the same surgical procedures except the LAD ligation. Functional and histological evaluations were performed 2 weeks after MI. Animals were anesthetized with 2% inhalant isoflurane and echocardiography was performed. After functional evaluation, mice were sacrificed with an overdose of pentobarbital (100 mg/kg) followed by cervical dislocation. Hearts were removed, transversely cut, and fixed in a formalin solution for preparation of heart sections. In addition, heart homogenates were prepared for western blot analysis and ELISA.

### Detection of ES Cell-Derived Cardiac Myocytes *In Vivo*

Tissue sections were deparaffinized and used for double immunolabeling to detect donor cells and cardiac myocytes. In brief, sections were incubated with Rabbit polyclonal anti-RFP antibody (1:500, Evrogen) to detect donor cells. After washing, the sections were incubated with goat anti-rabbit antibody (Invitrogen). Sections were then incubated with mouse anti-sarcomeric α-actin antibody (1:40, Sigma) for cardiac myocytes, and then sections were incubated with anti-mouse antibodies (M.O.M. kit, Vector Laboratories). Negative controls were used in each case by omitting primary or secondary antibody. Sections were mounted with Anti-fade Vectashield mounting medium containing 4,6-diamidino-2-phenylindole (DAPI; Vector Laboratories) to stain nuclei and examined with a Leica TCS SP2 laser scanning confocal microscope. For Notch-1 staining, sections were incubated with Notch-1 mouse monoclonal antibody (1:40, Abcam) using the M.O.M. kit (Vector Laboratories) in a humidified chamber.

### TUNEL Staining

Heart sections were deparaffinized and treated with proteinase K (25 µg/ml in 100 mM Tris-HCl). Cardiac myocyte apoptosis was detected using an in situ apoptotic cell death detection kit (Roche Applied Biosystems) and for nuclear visualization heart sections were also counterstained with 4',6-diamidino-2-phenylindole (DAPI; Vector Laboratories). Sections from each experimental group were examined by Olympus fluorescence microscopy. The percentage of total apoptotic nuclei was determined in the infarct and peri-infarct regions.

### Caspase-3 Activity Assay

Caspase-3 activity assay was performed using a caspase-3 colorimetric activity assay kit (cat #K106-200, BioVision) following the manufacturer’s instructions. Heart tissues were homogenized in a RIPA buffer containing protease inhibitor cocktail (Sigma) and then centrifuged at 14,000 x g for 5 min. Supernatant was collected and then transferred into new microfuge tubes. Protein assay was conducted using a Bio-Rad assay dye and colorimetric reaction for caspase-3 activity was measured at 405 nm in a Bio-Rad microtiter plate reader. Capsase-3 activity is represented as arbitrary units (A.U.).

### ELISA for pAkt and p-PTEN

The pAkt and p-PTEN activities were assayed using Phospho-Akt (PAN) ELISA kit (X1844k, Exalpha Biologicals) and Phospho-PTEN Sandwich ELISA kit (#7285, Cell Signaling), respectively, following the manufacturer’s instructions. In brief, heart samples were prepared and protein concentrations were quantified as described above. Standardized amount of samples were loaded to the wells (already coated with antibody) provided in the kit and incubated with detector antibody, HRP conjugate, substrate, and a stop solution with subsequent washings after each step. The color reaction was measured at 405 nm for pAkt activity and at 450 nm for p-PTEN activity in a Bio-Rad microplate reader. The optical density for each sample was normalized to the total proteins of each sample as determined by the Bradford assay.

### Echocardiography

Two weeks after MI, mouse heart function was evaluated by transthoracic echocardiography. Animals were anesthetized with 2% inhalant isoflurane using a nose cone system and echocardiography was performed using a Sonos 5500 Ultrasound system with a 15-6L hockey stick transducer. Left ventricular internal dimension-diastole (LVIDd), left ventricular internal dimension-systole (LVIDs), left ventricular volume at end diastole (EDV), left ventricular volume at end systole (ESV) were assessed in short axis view at the mid-papillary muscle level. Fractional shortening [FS = (LVIDd-LVIDs)/LVIDd x 100] and ejection fraction [EF = (EDV-ESV)/EDV x 100] were calculated for each experimental group.

### Statistical Analysis

Data are expressed as mean ± SE. Statistical analyses were performed using an unpaired t test and one-way ANOVA followed by Tukey test. A p value of <0.05 was considered significant.

## Results

### Genetic Modification of Mouse ES Cells with the Tβ4 Gene

**Figure 1:**
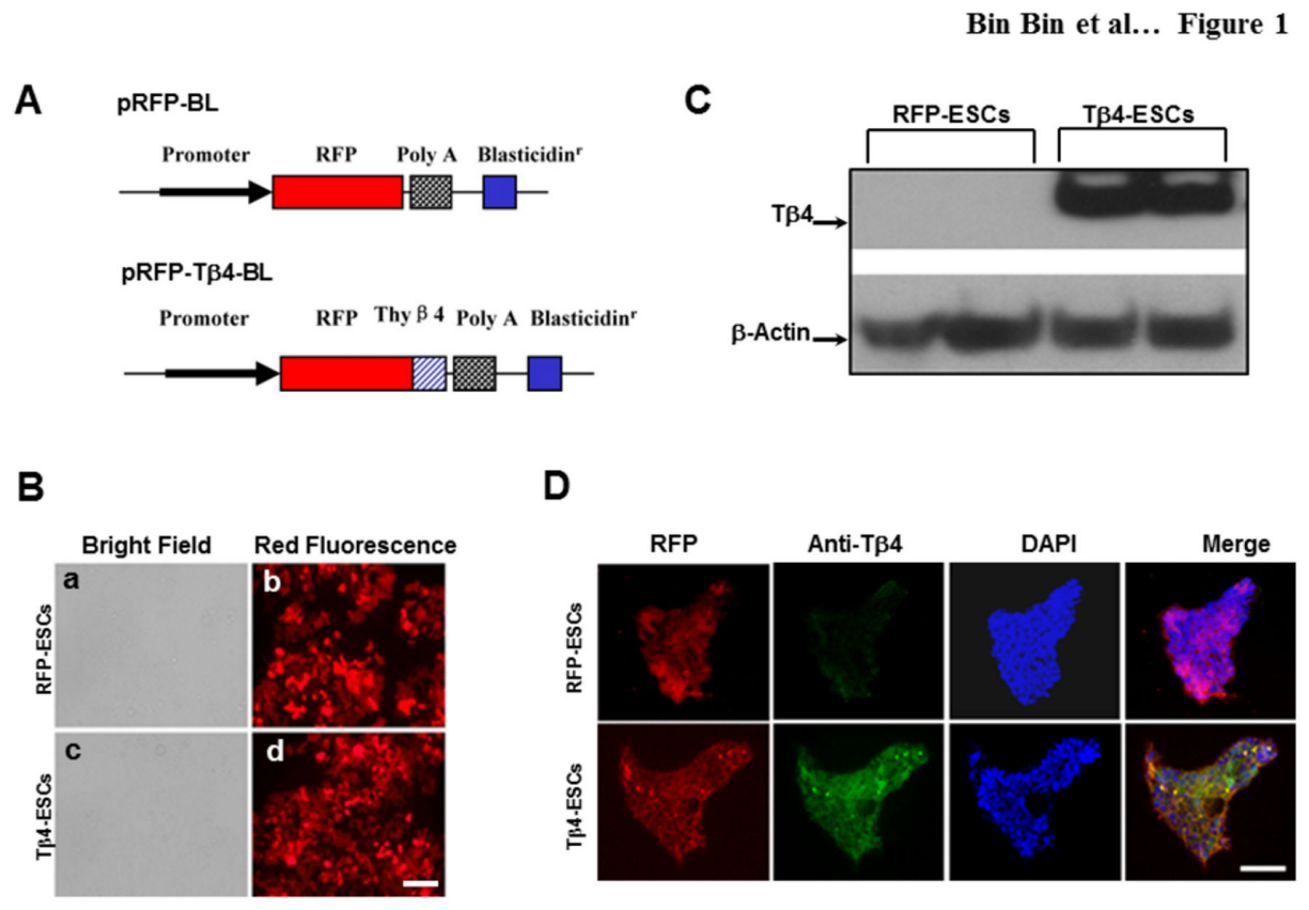
Expression Vector and Confirmation of Tβ4 Expression. **A**. Schematic drawing of the expression vectors, pRFP-BL and pRFP-Tβ4 –BL, for generation of stable ES cell lines. The cDNA coding for mouse Tβ4 was generated by PCR and cloned to the C-terminal of RFP gene in the same open reading frame. **B**. Fluorescent microscopy of red fluorescent protein expression in undifferentiated stable RFP-ES cells (b) and Tβ4-ES cells (d). Scale = 100µm. **C**. Western blot of mouse Tβ4 protein. **D**. Representative photomicrographs of Tβ4 expression in RFP-ES cells (top panels) and Tβ4-ES cells (bottom panels). RFP is shown in red, Tβ4 in green, nuclei in blue and merged images shown in far right panels. Scale = 200µm.

To examine the potential role of Tβ4 in the differentiation of ES cells into cardiac myocytes, we generated a stably transfected ES cell line overexpressing Tβ4. In Figure 1A, the top panel shows pRFP-BL, a control vector constructed to express RFP, whereas the bottom panel shows the expression construct of Tβ4 gene, pRFP-Tβ4-BL. Mouse ES cells were transfected with or without Tβ4 overexpressing vectors and cells were selected with 2 µg/ml of blasticidin. After 4 weeks of drug selection, two stable cell lines, RFP-ES cells, as a control, and Tβ4-ES cells were obtained (Figure 1B). In order to determine whether stably transfected Tβ4-ES cells have increased expression of Tβ4, western blot analysis was performed. Our data indicated a dramatic increase in the level of Tβ4 expression in Tβ4-ES cells, compared with the RFP-ES cells as a control (Figure 1C). Furthermore, we used immunocytochemistry and confocal microscopy to confirm the increased expression of Tβ4 in the Tβ4-ESCs (Figure 1D).

### Enhanced Differentiation *In vitro* of Tβ4-ES Cells into Cardiac Myocytes

**Figure 2:**
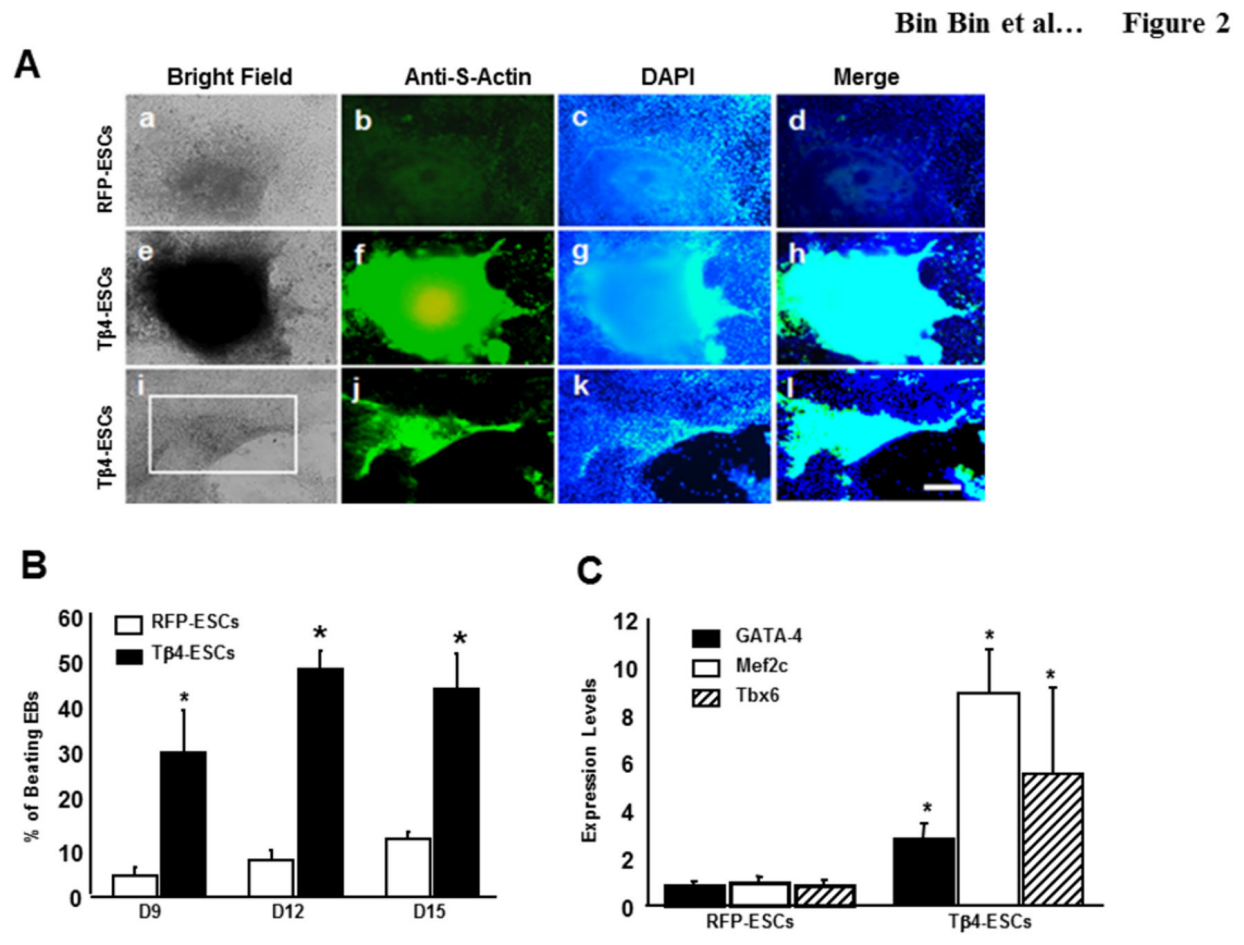
Effect of Tβ4 Expression on ES Cell Differentiation. **A**. Fluorescent microscopy of EBs derived from RFP-ES and Tβ4-ES cells. At D12 EBs were stained with anti- sarcomeric α-actin (S-actin) (green) and counterstained with DAPI for nuclear visualization (blue). The lower panel shows S-actin staining in a beating area (square box) in the EBs derived from Tβ4-ES cells. Scale = 200µm. **B**. Percentage of beating EBs during cardiac myocyte differentiation. Spontaneously beating EBs were examined and counted under a light microscope at D9, 12 and 15. **C**. Real-time PCR analysis of gene expression of GATA-4, Mef2c and Tbx6 at D12. Data are represented as mean ± SEM, *p< 0.05; vs. RFP ESCs.

To examine whether Tβ4 overexpression in ES cells has an effect on cardiac myocyte differentiation in the cell culture system, we generated EBs using the hanging drop method and examined morphological changes up to D15. There was no morphological difference observed between the EBs derived from RFP or Tβ4-ES cells up to D5. The spontaneously beating EBs were evident at D9 and were positively stained with sarcomeric α-actin (S-Actin), a cardiac myocyte specific marker, suggesting the presence of functional cardiac myocytes. Interestingly, we observed a markedly increased number of SA-positive areas in Tβ4 EBs, compared with the control RFP-EBs (Figure 2A). Moreover, the differentiating Tβ4-ES cells in the beating areas displayed a strong SA expression, as shown in the bottom panel of Figure 2A. Figure 2B shows a significant (p<0.05) increase in the number of beating EBs compared with RFP controls at D9; 30.8% vs. 4.4%; D12; 48.3% vs. 7.8%, and at D15; 44.2% vs. 12.2%. We also performed real-time PCR analysis to determine the expression levels of cardiac specific transcriptional factors such as GATA-4, Mef2c and Tbx6. The expression levels of GATA-4 were increased by 3 fold, Mef2c by 8 fold, and Tbx6 by 5 fold in Tβ4 EBs, compared with the RFP control (Figure 2C).

**Figure 3:**
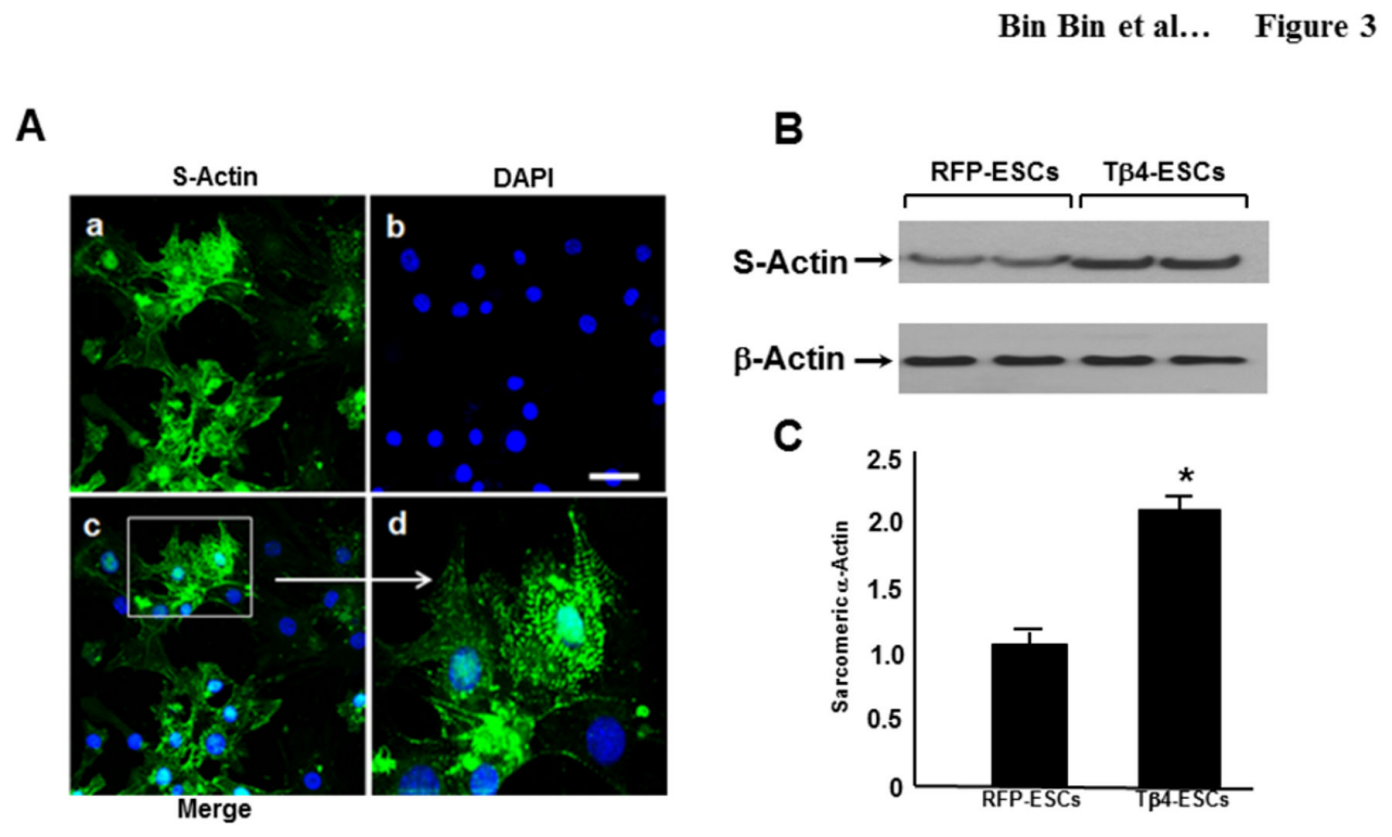
Tβ4-ES Cells Differentiate into Cardiac Myocytes. **A**. Confocal fluorescent microscopy of S-actin expression in Tβ4 EB-derived cardiac myocytes. Cells were stained with an anti-S-actin (a, green) and the nuclei in the same field were counterstained with DAPI (b, blue). Panel c shows the merged image for the overlay of S-actin and the nuclei. Panel d displays the image acquired at higher magnification (D, 400X). Scale = 25µm. **B**. Western blotting of S-actin expression in RFP-ESCs and Tβ4-ESCs. **C**. Quantitative analysis of S-actin expression. Data are represented as mean ± SEM, *p< 0.05; vs. RFP-ESCs.

We further examined whether or not Tβ4-ES-derived cardiac myocytes are positive for additional cardiac specific markers, such as S-actin. Figure 3A shows S-actin positive cells, which display green fluorescence whereas DAPI shows blue nuclei. A merged image of DAPI and S-actin, shown at high magnification, demonstrates the z-line staining cardiac myocyte-specific structural pattern (Figure 3A). We used western blot analysis to quantify the protein expression of cardiac specific S-Actin in beating EBs generated with or without Tβ4-ES cells (Figure 3B). The expression level of S-Actin was significantly (p<0.05) increased in Tβ4-EBs compared with control as determined by densitometry quantitative analysis (Figure 3C).

### Enhanced Cardiac Myocyte Differentiation in the Infarcted Heart following Transplantation of Tβ4-ES Cells

**Figure 4:**
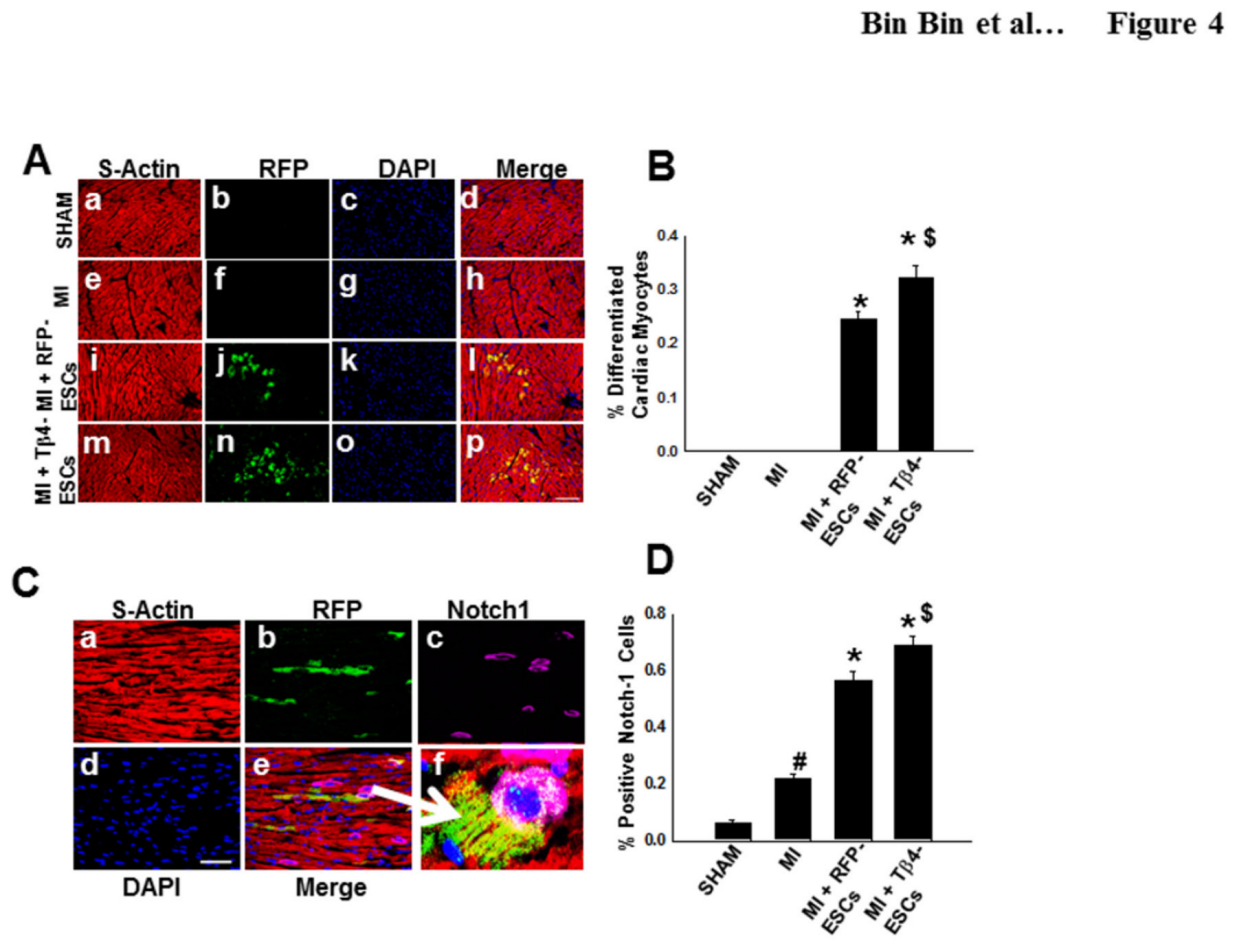
Effects of Tβ4 Expression on ES Cell Cardiac Myocyte Differentiation Post MI. **A**. Representative photomicrographs of immunostained tissue sections showing S-actin in red (a, e, i, m), RFP in green (b, f, j, n), dapi in blue (c, g, k, o) and merged images in pink (d, h, l, p). Scale = 50µm. **B**. Quantitative analysis shows a significant increase in cardiac myocyte differentiation in the Tβ4-ESC group. *p< vs. MI, #p<0.05 vs. RFP-ESCs. **C**. Representative photomicrographs showing S-actin in red (a), RFP in green (b), Notch1 in purple (c), dapi in blue (d), merged images in pink (e), and at high resolution (f). Scale=100µm. **D**. Histogram shows expression levels of Notch-1 in all groups. #p<0.05 vs. sham, *p<0.05 vs. MI, and $p<0.05 vs. RFP-ESCs.

In our cell culture system, we demonstrated that Tβ4 promoted cardiomyogenesis, as evidenced by immunofluorescent staining, western blot and real-time PCR for cardiac myocyte markers. Therefore, we hypothesized that transplantation of genetically modified Tβ4-ES cells into the heart would enhance cardiac myocyte differentiation *in vivo*. Accordingly, we performed double immunolabeling to detect donor ES cell-derived cardiac myocytes, using an anti-RFP antibody for donor cells and an anti-sarcomeric α-actin antibody for cardiac myocytes. As shown in Figure 4A, there were both RFP-positive and S-actin positive cells in the infarcted hearts transplanted with RFP-ES or Tβ4-ES cells, whereas there were no RFP-positive cells in the sham and MI groups. Interestingly, quantitative analysis further revealed that the number of ES cells-derived cardiac myocytes was significantly increased in the Tβ4-ES-transplanted heart following MI, compared with the RFP-ES-transplanted control, suggesting that transplanted Tβ4-ESCs enhance cardiac myocyte differentiation in the infarcted heart *in vivo* (Figure 4B).

### Notch Signaling Pathway Involving Cardiac Myocyte Differentiation

In Figure 4C images demonstrate S-actin in red (a), RFP-positive donor cells in green (b), Notch1-positive cells in purple (c), nuclei in blue (d), merged images (e), and high magnification of merged images (f). Our data suggests there is a significant increase in Notch-1 expression in newly differentiated cells in the hearts transplanted with RFP-ESCs and Tβ4-ESCs relative to the MI control (Figure 4D).

### Inhibition of Cardiac Myocyte Apoptosis in the Tβ4-ES Transplanted Hearts following MI

**Figure 5:**
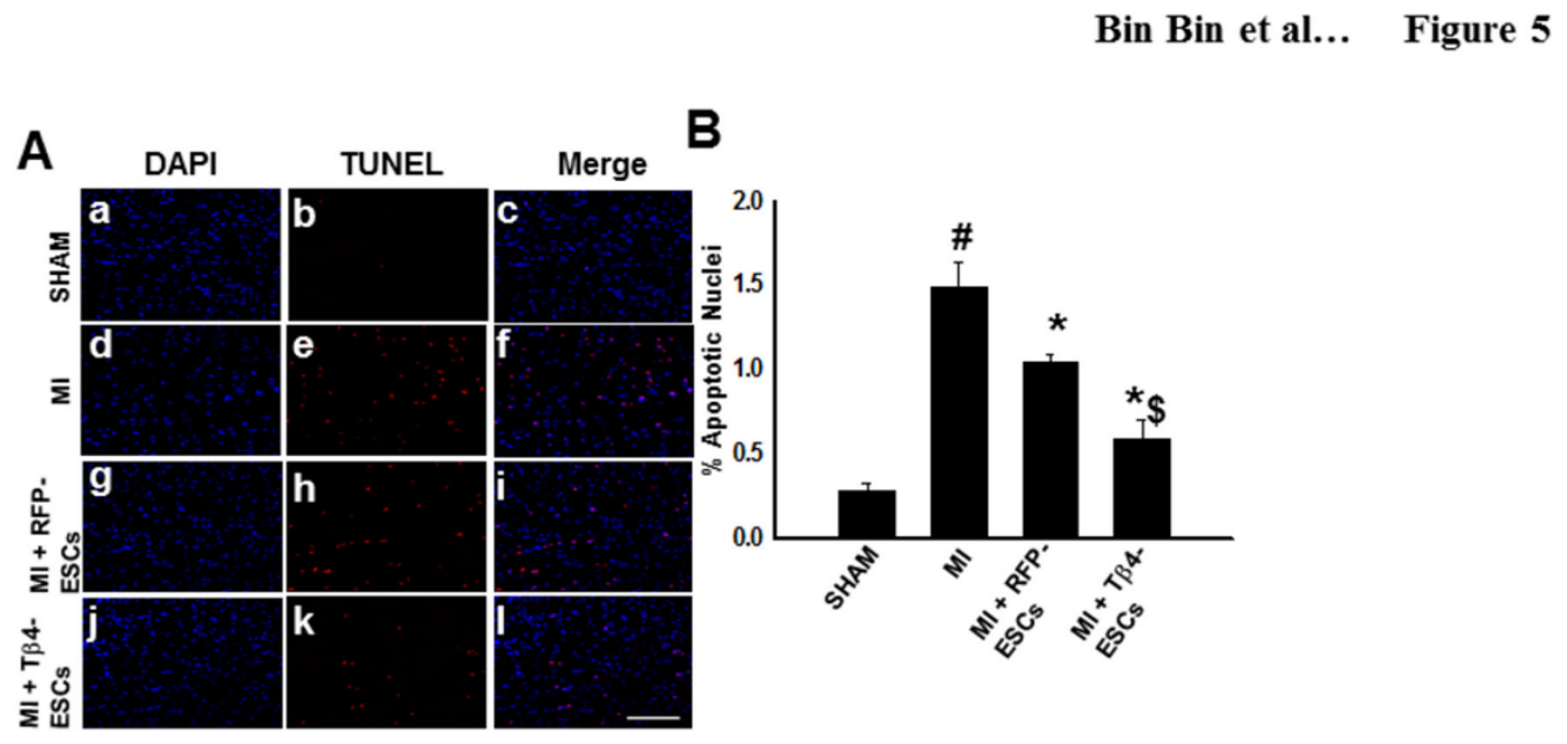
Transplanted Tβ4-ES Cells Inhibit Cardiac Myocyte Apoptosis in the Infarcted Heart. **A**. Representative photomicrographs of total nuclei stained with DAPI in blue (a, d, g, j), apoptotic nuclei stained with TUNEL in red (b, e, h, k), and merged nuclei in pink (c, f, i, l). Scale = 100µm. **B**. Quantitative analysis of apoptotic nuclei per tissue section. *p<0.01 vs. MI, #p<0.05 vs. sham, and $p<0.05 vs. RFP-ESCs.

**Figure 6:**
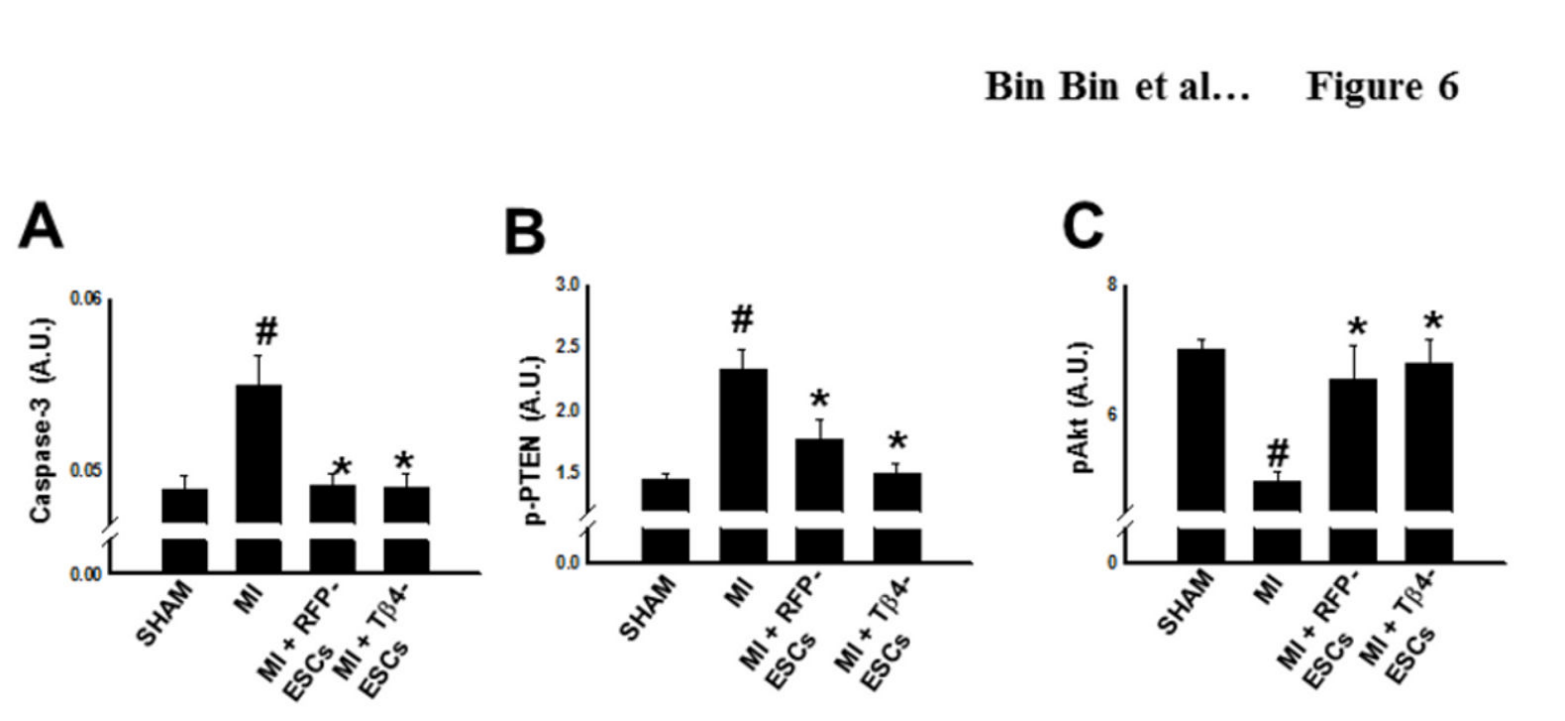
Effects of Tβ4 Expression on Caspase-3, pAkt, and p-PTEN Activities. Heart homogenates from each group were prepared for ELISA analysis of caspase-3, Akt, and p-PTEN. **A**. Quantitative analysis of caspase-3, **B**. p-PTEN, and **C**. pAkt activity in the hearts following cell transplantation. Data were represented as Mean ± SEM; *p<0.01 vs. MI, #p<0.05 vs. sham. n = 4-5 animals per group.

The effects of transplanted Tβ4-ES cells on cardiac myocyte apoptosis after MI were examined. Figure 5A shows the representative photomicrographs of TUNEL positive nuclei per heart section for both control and experimental groups. Quantitative analysis revealed a significant increase in apoptotic nuclei in the infarcted hearts relative to sham, whereas the number of apoptotic nuclei was markedly reduced in hearts transplanted with RFP-ESCs and Tβ4-ESCs compared to MI (Figure 5B). Notably, hearts transplanted with Tβ4-ESCs had a significantly lower number of apoptotic nuclei than the hearts injected with RFP-ES cells (Figure 5B), indicating that Tβ4 is a potential pro-survival factor that inhibits the MI-induced cardiac myocyte apoptosis. Furthermore, there was a significant decrease in caspase-3 activity in hearts transplanted with either Tβ4-ES cells or RFP-ES cells, compared to the infarcted hearts without cell transplantation (p<0.05, Figure 6A). However, there was no statistical significance in caspase-3 activity between Tβ4-ES cell and RFP-ES cell groups.

Multiple signaling pathways contribute to apoptotic inhibition, including the well-known PI3K/Akt survival pathway. To examine whether inhibition of PTEN, a negative regulator of the Akt pathway, is implicated in cardioprotection of Tβ4-ES cells against MI, we performed ELISA for phospho-PTEN. It was found that hearts transplanted with either Tβ4-ES cells or RFP-ES cells had a significant reduction in PTEN expression compared with the MI group, but there was no significant difference between the cell-transplanted heart groups (Figure 6B). Additionally, our quantitative analysis suggests Akt expression was significantly augmented following transplantation of RFP-ESCs and Tβ4-ESCs post-MI relative to MI controls (Figure 6C).

### Reduced Cardiac Fibrosis in the Tβ4-ESC Transplanted Hearts following MI

**Figure 7:**
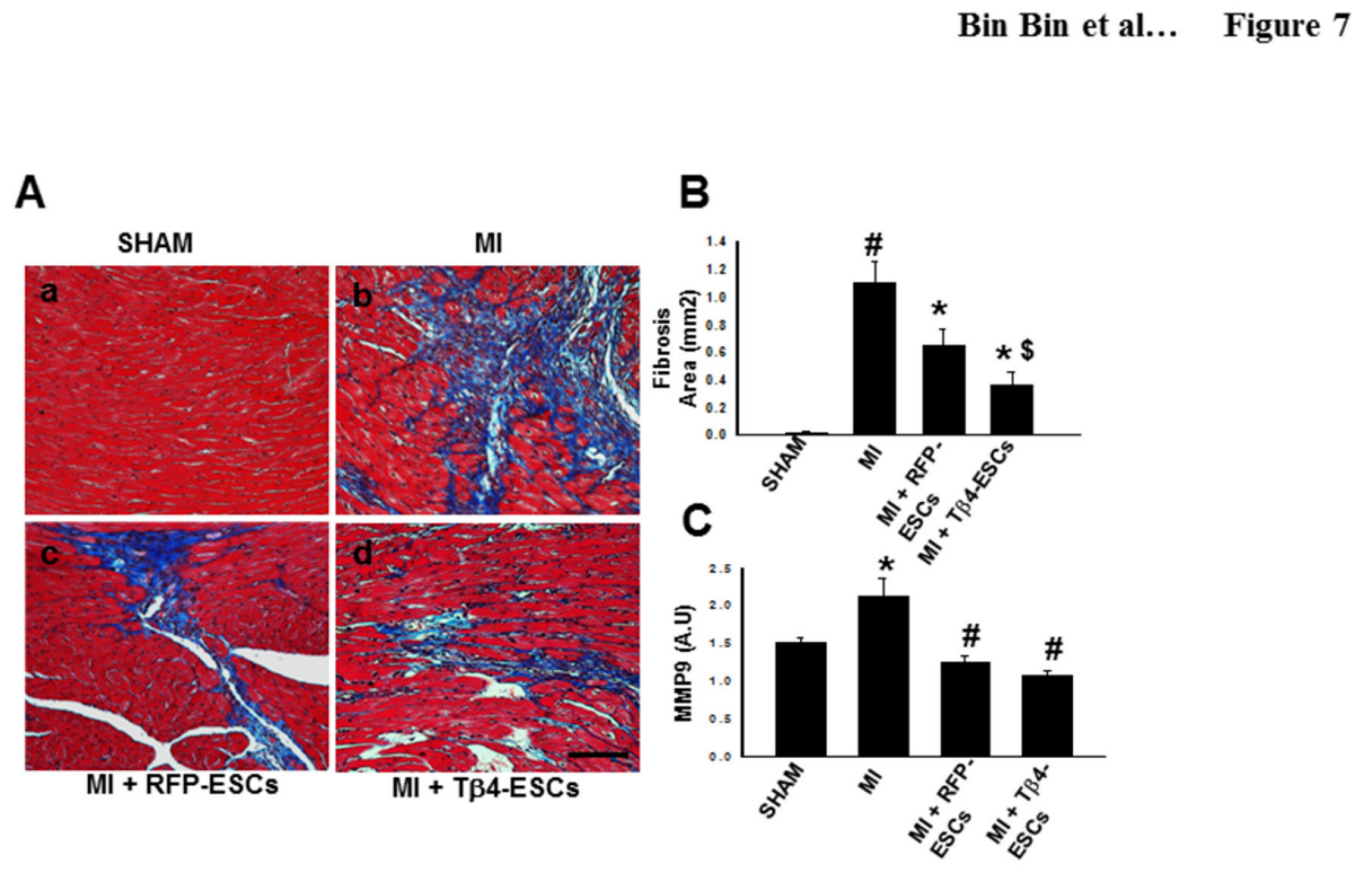
Transplanted Tβ4-ES Cells Reduce Cardiac Fibrosis in the Infarcted Mouse Heart. **A**. Representative photomicrographs of tissue sections stained with Masson’s trichrome at D14 post MI surgery. Scale =100µm. **B**. Quantitative analysis of interstitial fibrosis for control and experimental groups. #p<0.05 vs. sham, *p<0.05 vs. MI, and $p<0.05 vs. RFP-ESCs. **C**. Histogram illustrates quantitative MMP-9 expression. #p<0.05 vs sham, *p<0.05 vs. MI. n = 5-7 animals per group.

To examine the effects of transplanted Tβ4 on interstitial fibrosis, we stained heart sections with Masson’s trichrome (Figure 7A) and quantitated the collagen-positive fibrotic areas using NIH ImageJ software. Quantitative data analysis demonstrated that both transplanted Tβ4-ES and RFP-ES cells significantly reduced the amount of fibrosis in the infarcted hearts at day 14 after MI (Figure 7B). Importantly, hearts transplanted with Tβ4-ESCs had a further significant reduction in cardiac fibrosis, compared with the RFP-ESC group (p<0.05, Figure 7B), suggesting that genetic modification of ES cells with Tβ4 is a novel therapeutic strategy to attenuate cardiac remodeling following MI.

To elucidate mechanisms by which RFP-ES and Tβ4-ES cells inhibit fibrosis post-MI, levels of MMP-9, a pro-fibrotic modulator, were examined. Our data suggested a significant reduction in MMP-9 expression following MI in RFP-ES and Tβ4-ES cell transplanted hearts compared to MI hearts (Figure 7C). However, statistical significance was not obtained between RFP-ESC and Tβ4-ESC groups (Figure 7C).

### Improved Cardiac Function in the Infarcted Hearts following Transplantation of Tβ4-ES Cells

**Figure 8:**
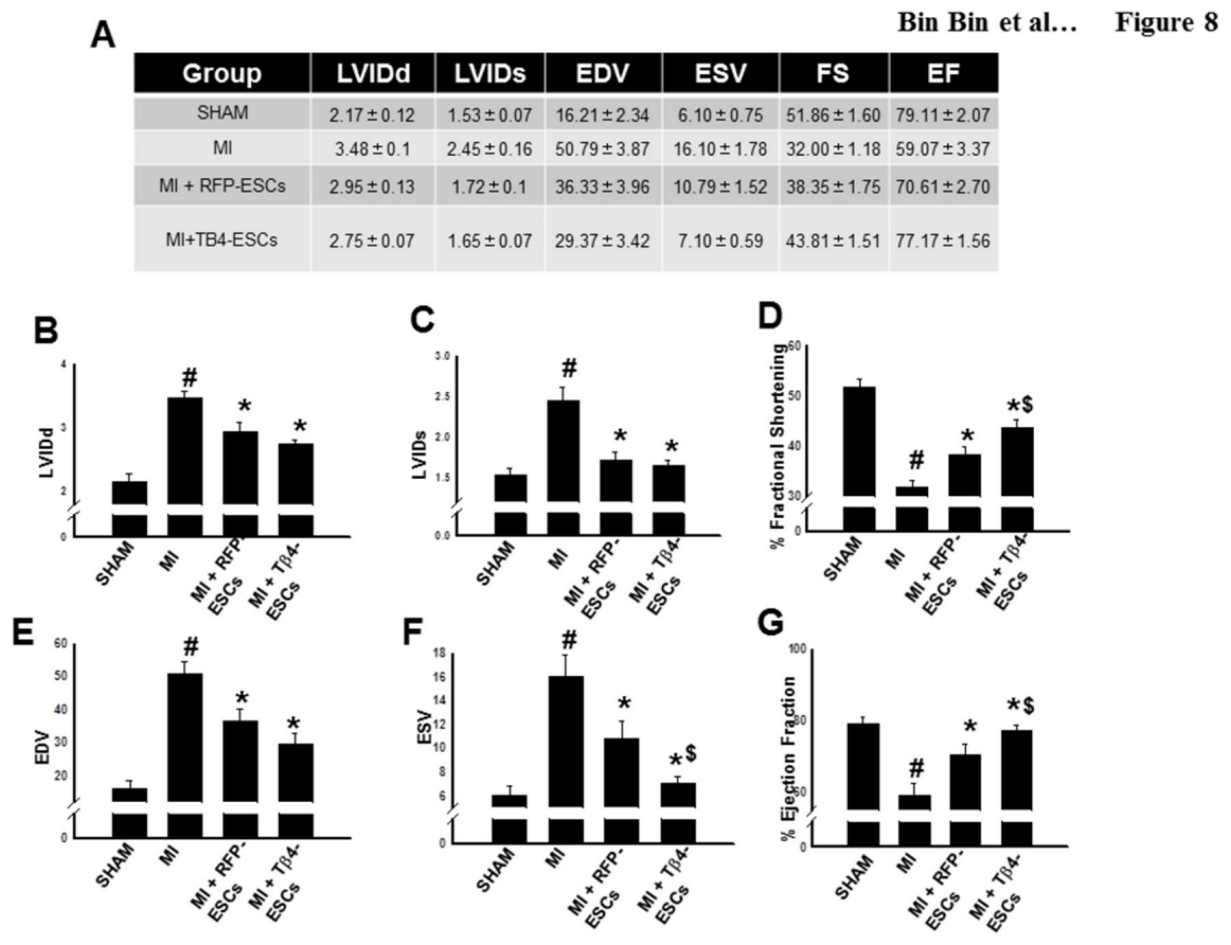
Transplanted Tβ4-ES Cells Improve Cardiac Function in the Infarcted Heart. Echocardiography was performed D14 following MI. **A**. Raw functional data. Histograms show average quantified measurements of **B**. left ventricular internal diameter during diastole (LVIDd) **C**. left ventricular internal diameter during systole (LVIDs) **D**. fractional shortening FS% **E**. end diastolic volume (EDV) **F**. end systolic volume (ESV) **G**. and ejection fraction EF% at 2 weeks after MI for all treatment groups. #p<0.05 vs. sham, *p<0.05 vs. MI, and $p<0.05 vs. RFP-ESCs. Data set are from n=6-8 animals/group.

We performed echocardiography to evaluate the effects of transplanted Tβ4-ES cells on cardiac function 2 weeks after MI. As shown in Figure 8, there was a significant (p<0.05) decrease in both fractional shortening and ejection fraction in the infarcted mouse hearts. Transplanted ES cells expressing RFP significantly improve cardiac function following MI (Figure 8D). Interestingly, transplanted Tβ4-ES cells further increased fractional shortening in the infarcted hearts, compared with the RFP-ES cells (Mean ± SEM, Tβ4-ESCs: 43.81±1.51 vs. RFP-ESCs: 38.35±1.75, p<0.05, Figure 8D) and ejection fraction (Mean ± SEM, Tβ4-ESCs: 77.17±1.56 vs. RFP-ESCs: 70.61±2.70, p<0.05, Figure 8G), suggesting that genetic modification of ES cells with Tβ4 produces more beneficial effects on cardiac function following MI.

## Discussion

Stem cell therapy is emerging as a novel therapeutic approach to treat various heart diseases including myocardial infarction. Increasing evidence from embryonic and adult stem cell transplantation studies demonstrate that transplanted stem cells significantly improve cardiac function, which is likely associated with the inhibition of cardiac apoptosis and fibrosis and generation of new cardiac myocytes [23,26-28]. However, the amount of differentiated cardiac myocytes following transplantation is very limited. Identification of new approaches to enhance cardiac myocyte differentiation from ES cells would bring researchers a step closer to optimizing cell therapy for clinical applications. To date, several strategies for inducing differentiation of stem cells into cardiac myocytes have been reported including the use of dimethysulfoxide [23], transforming growth factor-β [9], ascorbic acid [11], retinoic acid [29], 5-Aza-2’deoxycytidine [30], and bone morphogenic protein-2 [22].

Tβ4, a small acidic actin-sequestering peptide, participates in many important cellular events including inhibition of apoptosis and angiogenesis in the heart [15-18]. It has been suggested that Tβ4 is a novel signaling molecule, which activates various cell type differentiation [15,16,21,31]. However, its role in the differentiation of ES or adult stem cells into cardiac myocytes has not been examined. Therefore, we designed the present study to determine whether overexpression of Tβ4 in ES cells would enhance cardiac myocyte differentiation in the cell culture system and *in vivo*. To the best of our knowledge, this is the first report on the evaluation of the effects of Tβ4 on cardiac myocyte differentiation from genetically modified ES cells.

In the present study, we established two stably transfected cell lines, RFP-ES and Tβ4-ES cells, and observed no effect on cell proliferation, nor significant changes in morphology in the EBs, compared with untransfected ES cells (data not shown). We observed beating EBs derived from stably transfected RFP-ES cells at D9 and this was further enhanced at D12 and D15. Our data is in accordance with earlier published studies suggesting that cardiac myocyte beating generally occurs after D7 [10,32-34]. Interestingly, Tβ4-ES cells had a significant increase in the number of beating EBs, compared with RFP-ES cells. We further confirmed the presence of cardiac myocytes in the beating EBs using immunofluorescent staining as well as western blot data for S-actin. Similarly, It has been reported that Tβ4 plays a significant role in the heart development at embryonic stage (E10) and at E14.5 because expression of Tβ4 was widely observed in the heart [16]. Our cell culture data demonstrating increased beating cardiac myocytes derived from Tβ4-EBs at D9 is in the agreement of the role played by Tβ4 in the developing heart. Furthermore, our real time PCR data from Tβ4 derived EBs revealed significant up regulation of mRNA levels of cardiac transcription factors, GATA-4, Mef2c and Tbx6, suggesting a possible role of up regulating these early transcriptional factors in cardiac myocyte differentiation which is in accordance with previous data [35-37].

Based on our cell culture studies we hypothesized that transplanted Tβ4-ESCs would promote cardiac myocyte differentiation and provide indigenous cardioprotection, thus protecting left ventricular function in the post-MI heart. Our data suggests that Tβ4-ESCs generate significant numbers of new cardiac myocytes in the injured heart compared to all other experimental and control groups (Figure 4B). Similarly, pretreatment of mice with Tβ4 peptide promoted cardiac repair, which was attributed to the differentiation of epicardial cells into cardiac myocytes [16]. However, the administration of Tβ4 after MI did not reprogram epicardial cells into cardiac myocytes [16]. Moreover, the native Tβ4 peptide has been the subject of a clinical trial for the treatment of MI [38]. The current study did not investigate the effects of Tβ4-ES cells on epicardial cell differentiation but suggest that genetically modified Tβ4-ESCs may be more advantageous for cardiac myocyte regeneration in the clinical setting as they can directly differentiate themselves into cardiac myocytes.

The Notch pathway plays a pivotal role in a myriad of cardiac developmental and pathological scenarios, including regulation of cardiac progenitor cell fate and the proliferation of cardiac myocytes [24,25,39]. In the present study, we demonstrate that transplanted Tβ4-ESCs differentiate into cardiac myocytes and these newly formed cells have increased expression of Notch-1 in the post-MI heart, suggesting Notch-1 is also regulated in the derivation of cardiac myocytes from the transplanted cells. However, previous studies have suggested transient activation of Notch-1 following cardiac assault promotes myocardial repair and regeneration [24]. Moreover, we acknowledge the interrelationship of the Notch-1 pathway and other reparative and protective pathways occur during myocardial injury, which are complex and multifaceted. Identification of these associations between Notch-1 signaling molecules and Tβ4-ESCs is well beyond the scope of the current study.

Additionally, we have examined whether transplanted Tβ4-ES cells enhance cardiac repair through additional protective mechanisms, including inhibition of cardiac myocyte apoptosis following MI. It has been reported that Tβ4 reduces cardiac myocyte death in the cell culture system after hypoxia [20] and also in the adult mouse heart after MI, regardless of intramyocardial or intraperitoneal systemic application [15]. Consistent with previous studies, we have found that transplantation of unmodified ES cell reduces to some extent cardiac myocyte apoptosis in the MI model [8,9]. In particular, transplanted Tβ4-ES cells provided significantly greater protection against cardiac myocyte apoptosis in the infarcted heart than RFP-ES cells.

In an attempt to define mechanisms promoted by transplanted Tβ4-ES cells, which govern apoptosis following MI, we investigated the potential correlation between Tβ4-ESCs and the Akt pathway. Widely accepted, the Akt signaling pathway plays a pivotal role in a host of cellular processes including growth, proliferation, and survival. Phosphatase and tensin homolog (PTEN) inhibits PI3K phosphorylation and subsequent phosphorylation and activation of Akt. Our data suggests a significant increase in PTEN activity following MI, which is corroborated in other myocardial injury studies [23,40]. However, following transplantation of either Tβ4-ES cells or RFP-ES cells, we report a significant decrease in PTEN expression, compared with the MI group. Based on these findings, we hypothesized that downregulation of PTEN would lead to increased Akt activity, which would account for the diminished cardiac myocyte apoptosis and abrogated adverse cardiac remodeling documented within the current study. Therefore, our assessed levels of Akt by ELISA demonstrate significant upregulation in Akt activity in hearts transplanted with either Tβ4-ES cells or RFP-ES cells. In accordance with our findings, a recent report has demonstrated that administration of Tβ4 promotes cardiac myocyte survival through activation of Akt in a mouse MI model [15]. Ultimately, our data suggest that Tβ4-ES cells enhance cardioprotection against MI at least in part through inhibition of apoptosis and activation of the Akt pathway. Whether the enhanced cardiomyogenesis observed in Tβ4-ES cell-transplanted hearts is consequent to augmentations to PTEN/Akt pathway was not addressed within the context of the current study and will require future investigation.

Moreover, cardiac myocyte apoptosis and fibrosis formation contribute significantly to the pathological cardiac remodeling following MI [23]. A dramatic loss of cardiac myocytes in the infarcted heart results in subsequent replacement and proliferation of non-myocytes, such as cardiac fibroblasts, forming a fibrotic network to rescue the architecture of injured myocardium. This process involves metalloproteinase (MMP) activation, extracellular matrix (ECM) degradation and collagen deposition in the infarcted heart, leading to interstitial and vascular fibrosis, scar formation, and eventual cardiac dysfunction. In agreement to the previous studies [8], transplanted ES cells (Tβ4-ES or RFP-ES cells) inhibit the formation of fibrosis in the MI model. Furthermore, there is a significant decrease in fibrosis in the infarcted heart transplanted with Tβ4-ES cells, compared with the RFP-ES cell group. Although the underlying mechanism remains to be examined, our data clearly indicates that ES cells overexpressing Tβ4 can produce anti-fibrotic effects mediated through MMP-9 inactivation following MI.

Finally, we examined the effects perpetrated by our Tβ4-ESCs on cardiac function following MI. For the first time, we have found that transplanted ES cells overexpressing Tβ4 significantly improve cardiac function 2 weeks after MI, as demonstrated by increased fractional shortening and ejection fraction. We suggest that enhanced left ventricular output following transplantation of Tβ4-ESCs is indicative of enhanced cardiac myocyte differentiation and augmented cardioprotection provided by these cells in the injured myocardium.

In summary, we have examined the effects of Tβ4 on cardiac myocyte differentiation and cardioprotection using genetically modified ES cells. Our data collectively suggests 1) Tβ4 overexpression enhances differentiation of mouse ES cells into functional cardiac myocytes *in vitro* and *in vivo*, 2) Tβ4-ESCs inhibit apoptosis through down-regulation of PTEN and upregulation of Akt in the infarcted myocardium, 3) transplantation of Tβ4-ESCs reduce fibrosis formation via inhibition of MMP-9 activation following MI, and 4) enhanced left ventricular function is obtained following transplantation of Tβ4-ESCs post-MI. It is suggested that transplantation of genetically modified Tβ4-ES cells into the injured heart is a novel therapeutic approach, which provides additional differentiation potential and enhanced cardioprotection against MI, relative to unmodified ES cells.
